# Generation *in vivo *of peptide-specific cytotoxic T cells and presence of regulatory T cells during vaccination with hTERT (class I and II) peptide-pulsed DCs

**DOI:** 10.1186/1479-5876-7-18

**Published:** 2009-03-19

**Authors:** Mark M Aloysius, Alastair J Mc Kechnie, Richard A Robins, Chandan Verma, Jennifer M Eremin, Farzin Farzaneh, Nagy A Habib, Joti Bhalla, Nicola R Hardwick, Sukchai Satthaporn, Thiagarajan Sreenivasan, Mohammed El-Sheemy, Oleg Eremin

**Affiliations:** 1 Section of Surgery, Biomedical Research Unit, Nottingham Digestive Diseases Centre, University of Nottingham, NG7 2UH, UK; 2 Institute of Infection and Immunity, School of Molecular Medical Sciences, Nottingham University Hospitals, University of Nottingham, NG7 2UH, UK; 3 Lincolnshire Oncology Centre, Lincoln County Hospital, Lincoln, LN2 5QY, UK; 4 Research and Development Department, Lincoln County Hospital, Lincoln, LN2 5QY, UK; 5 Department of Haematological & Molecular Medicine, Rayne Institute, King's College, 123 Cold Harbour Lane, London, SE5 9NU, UK; 6 Section of Surgery, Department of Surgical Oncology and Technology, Imperial College London, Du Cane Road, London, W12 0NN, UK

## Abstract

**Background:**

Optimal techniques for DC generation for immunotherapy in cancer are yet to be established. Study aims were to evaluate: *(i) *DC activation/maturation milieu (TNF-α +/- IFN-α) and its effects on CD8+ hTERT-specific T cell responses to class I epitopes (p540 or p865), *(ii) *CD8+ hTERT-specific T cell responses elicited by vaccination with class I alone or both class I and II epitope (p766 and p672)-pulsed DCs, prepared without IFN-α, *(iii) *association between circulating T regulatory cells (Tregs) and clinical responses.

**Methods:**

Autologous DCs were generated from 10 patients (HLA-0201) with advanced cancer by culturing CD14+ blood monocytes in the presence of GM-CSF and IL-4 supplemented with TNF-α [DCT] or TNF-α and IFN-α [DCTI]. The capacity of the DCs to induce functional CD8+ T cell responses to hTERT HLA-0201 restricted nonapeptides was assessed by MHC tetramer binding and peptide-specific cytotoxicity. Each DC preparation (DCT or DCTI) was pulsed with only one type of hTERT peptide (p540 or p865) and both preparations were injected into separate lymph node draining regions every 2–3 weeks. This vaccination design enabled comparison of efficacy between DCT and DCTI in generating hTERT peptide specific CD8+ T cells and comparison of class I hTERT peptide (p540 or p865)-loaded DCT with or without class II cognate help (p766 and p672) in 6 patients. T regulatory cells were evaluated in 8 patients.

**Results:**

*(i) *DCTIs and DCTs, pulsed with hTERT peptides, were comparable (p = 0.45, t-test) in inducing peptide-specific CD8+ T cell responses. *(ii) *Class II cognate help, significantly enhanced (p < 0.05, t-test) peptide-specific CD8+T cell responses, compared with class I pulsed DCs alone. *(iii) *Clinical responders had significantly lower (p < 0.05, Mann-Whitney U test) T regs, compared with non-responders. 4/16 patients experienced partial but transient clinical responses during vaccination. Vaccination was well tolerated with minimal toxicity.

**Conclusion:**

Addition of IFN-α to *ex vivo *monocyte-derived DCs, did not significantly enhance peptide-specific T cell responses *in vivo*, compared with TNF-α alone. Class II cognate help significantly augments peptide-specific T cell responses. Clinically favourable responses were seen in patients with low levels of circulating T regs.

## Introduction

Induction of an effective anti-tumour response requires the active and integrated participation of host dendritic cells (DCs), taking up tumour-associated antigens (TAAgs) and generating Ag-specific T cells[1]. The transition of DCs from Ag-processing to Ag-presenting cells is accompanied by increased expression of class I and II major histocompatibility (MHC) proteins, CD80 and CD86 co-stimulatory molecules and CD40 adhesion molecules. These changes enhance the ability of DCs to present Ag to naïve T lymphocytes in secondary lymphoid compartments and, thereby, generate TAAg-specific cytotoxic T lymphocytes (CTLs). Activated and mature DCs produce a range of cytokines, notably interleukin-12 (IL-12), which stimulates CD4+ T helper 1 (Th1) cell activation and development[2]. Strategies for exploiting DCs to induce T cell responses to tumours have used both *in vivo *and *ex vivo *approaches in humans[1].

### DC maturation and activation milieu

The generation of DCs from peripheral blood can be achieved using a variety of maturation factors [3-8]. Purified CD14+ monocytes cultured with granulocyte macrophage-colony stimulating factor (GM-CSF) and IL-4 have been used most frequently in clinical trials, to date [1,9]. Culturing blood monocytes in the presence of IL-4 and GM-CSF is an efficient method to obtain large numbers of DCs. However, these DCs exhibit an immature phenotype (CD40 low/intermediate, CD86 low/intermediate and CD1a high) [10-12]. Thus, additional factors are needed to facilitate optimal activation and maturation of the cells *in vitro*.

Tumour necrosis factor-alpha (TNF-α) has been shown to be a crucial inflammatory maturation factor that prevents CD14+monocytes differentiating into macrophages and drives them along the DC differentiation pathway[13]. TNF-α has also been recently shown to enhance survival of *ex vivo *cultured DCs by inhibition of apoptosis [14]. Evidence is emerging that TNF-α matures DCs to the CD70+ phenotype which is crucial for activating CD4+T cells driving a Th1 response capable of augmenting CD8+ CTL responses [15-17]. TNF-α, therefore, has been used to induce the maturation of DCs following a period of expansion and differentiation of CD34+ or CD14+ monocytes, as part of a cocktail of cytokines. Furthermore, DCs engineered to express TNF-α maintain their maturation status and induce more efficient anti-tumour immune responses[18]. Thus, TNF-α has been used in large scale production of DCs for immunotherapy studies in humans [19,20].

Interferon-alpha (IFN-α) is a potent immunoregulatory cytokine, secreted early during the immune response by monocytes/macrophages and other cells [21,22]. Type I IFN is emerging as an important signal for differentiation and maturation of DCs [23-27]. In the presence of GM-CSF and IFN-α, monocytes are capable of differentiating into IFN-DCs[28]. IFN-DCs show the phenotypical and functional properties of partially mature DCs[28]. Such DCs have the capacity to induce Th1 responses and to promote efficiently *in vitro *and *in vivo *the expansion of CD8+ T lymphocytes [29]. Although all these studies have invariably used IFN-α and GM-CSF (without IL-4) to generate their IFN-DCs, there are no clinical studies published, to date, using the combination of GM-CSF, IL-4, TNF-α ± IFN-α to generate DCs for immunotherapeutic purposes. However, the effect of IFN-α on the optimal maturation and generation of monocyte-derived DCs with consequent induction of optimal and maximal anti-tumour CD8+ CTLs in patients with cancer, has yet to be established. There has also been some conflicting evidence as regards the function of IFN-α matured DCs [30,31].

Jonuleit's cocktail of TNF-α, IL-1, IL-6 and prostaglandin E2 (PGE_2_) for maturing DCs, has been, until recently, regarded as the gold standard for optimally maturing monocyte-derived DCs [32]. However, recent studies have shown that PGE_2 _in this cocktail rendered monocyte-derived DCs resistant to *in vivo *licensing by costimulatory molecules, such as CD40, and failed to induce IL-12 but produced the immune suppressive factor IL-10 [33,34]. Moreover, DCs matured with Jonuleit's cocktail have been shown to promote the expansion of CD4+CD25+ *foxp3 *high, T regulatory cells (Tregs) [35]. This was the rationale for choosing to compare TNF-α by itself or in combination with IFN-α as a maturation and activation factor for *ex vivo *monocyte-derived DCs, instead of the standard Jonuleit's DC maturation cocktail. Our previous work *in vitro *had demonstrated that monocyte-derived DCs matured with TNF-α and IFN-α were phenotypically and functionally superior to DCs matured with TNF-α alone[36].

The **first aim **of our study, therefore, was to evaluate and compare the efficacy of two different cytokine DC-maturation and activation factors [TNF-α (DCT) vs. TNF-α+IFN-α (DCTI)] for *ex vivo *generation of DCs from

CD 14+ monocytes activated with GM-CSF and IL-4. We compared hTERT-specific CD8+T cell responses elicited *in vivo *between the above two DC preparations. In our previously published work we had shown that this cytokine combination (GM-CSF, IL-4, TNF-α ± IFN-α) was capable of generating DCs *in vitro *from CD14+ monocytes obtained from healthy individuals and patients with cancer[36]. These DCs were activated but relatively immature, strongly phagocytic and induced CD8+T cell responses *in vitro*. The approach we used recognized that IFN-α is a potent cytokine inducing the maturation of DCs [26]. IFN-α, however, fails to terminally mature monocyte-derived DCs, which is a great advantage in immunotherapy where antigen uptake and processing following peptide pulsing of the DCs is required before they can be used to vaccinate patients[37,38].

### Human telomerase reverse transcriptase (hTERT)

hTERT is expressed in >85% of human tumours, and can be regarded as a putative TAAg [39]. Two HLA-A2 binding hTERT peptides, p540 and p865, are known to be immunogenic *in vitro *[40]. DCs pulsed with p540 were also able to induce tetramer positive T cell responses (detectable after further *in vitro *stimulation) when injected into patients with a variety of cancers [41,42]. In our study, autologous DC vaccines were prepared with and without INF-α, and each pulsed with a different hTERT peptide, and administered simultaneously to separate lymph node draining areas in the limbs. We evaluated our vaccination protocols, using a previously well described design for comparing two different DC preparations in the same patient [43]. Peptide-specific MHC tetramer analysis was used to track differential T cell responses to each vaccine, allowing direct comparison of the *in vivo *function of both vaccines in each patient. We adapted this study design further to compare DCT vaccines pulsed with class I epitope of hTERT, with or without class II epitopes. This strategy has been used previously with melanoma-related antigen class I peptides to compare the activity of immature and mature DCs [43].

The **second aim **of our study was to evaluate the ability of DC preparations (DCT) pulsed with class I (p540 or p865) and II (p766 and p672) epitopes of hTERT, to generate an enhanced hTERT-specific CD8+CTL response, compared with using class I epitopes alone. CD4+ cognate help generated by DCs pulsed with class II peptides has been shown to be crucial to maintain the levels of CD8+T cells in the circulation, through augmentation of T memory cell responses [44,45]. However, there are no published studies on the use of class II cognate helper peptides, with class I peptides of hTERT.

### T regulatory cells

In mice, high levels of circulating Tregs are associated with poor anti-cancer therapeutic responses [46-48]. T regs are known to inhibit activation of CD8+ T cells and NK (natural killer) cells [49]. In humans, the reduced efficacy of cell-mediated immunity as a result of ageing has been attributed to concurrent enhancement of circulating Tregs [49]. In clinical studies, reduction of circulating T regs by chemotherapeutic agents has resulted in enhanced therapeutic anti-cancer responses [50,51]. However, there are no studies published, to date, on T regs in the circulation of patients undergoing hTERT-based immunotherapy and no relationship has been established with clinical responses.

The **third aim **of our study, therefore, was to evaluate the levels of circulating T regs (CD4+CD25+*foxp3 *high phenotypic profile) in patients undergoing vaccination and to establish any association with clinical responses.

In summary, we have employed a novel immunization strategy in patients with advanced cancer by using two different DC maturation processes (10 patients) and two different DC peptide pulsing protocols (6 patients). We have been able to document the enhanced generation of functional peptide-specific CD8+ T cells, readily detectable *ex vivo *without further re-stimulation *in vitro*. T reg levels were also documented in vaccinees (8 patients); very low levels were associated with partial clinical responses. hTERT vaccination was safe and well tolerated. The results obtained in our study are novel and have not been previously published, and are very relevant to the future development of effective anti-cancer immunotherapy.

## Materials and methods

### Trial Eligibility

Ethical approval for vaccination of patients with advanced cancer using DCs pulsed with synthetic peptides of hTERT was obtained from the Lincolnshire Research Ethics Committee. Approval for the use of GMP grade hTERT peptides and cytokines was obtained from the Medicines and Healthcare Products Regulatory Agency (MHRA), UK. Patients attending the United Lincolnshire Hospitals NHS Trust, with proven advanced and progressive malignant disease, with no further effective anti-cancer therapeutic option available, were invited to participate. HLA-0201 +ve, Hepatitis B&C -ve, HIV-ve patients were assessed for suitability for the study. All patients had a WHO performance status of 2 or better. Women were either post-menopausal or using suitable contraception. Patients were not taking systemic steroids, nor did they have any medical contraindication to enrolment.

### Patients

Ten patients (6 with prostate cancer, 2 with malignant melanoma, 1 with breast cancer and 1 with lung cancer) were enrolled into the 1^st ^phase of the study (A), which was to compare DCT with DCTI. The 2^nd ^phase of the study (B) enrolled 6 patients (3 with prostate cancer, 1 with colorectal cancer, 1 renal cancer and 1 head and neck cancer) and compared class I+II hTERT peptide-pulsed DCTs with class I hTERT peptide-pulsed DCTs alone.

### Trial Design

**Figure 1:**
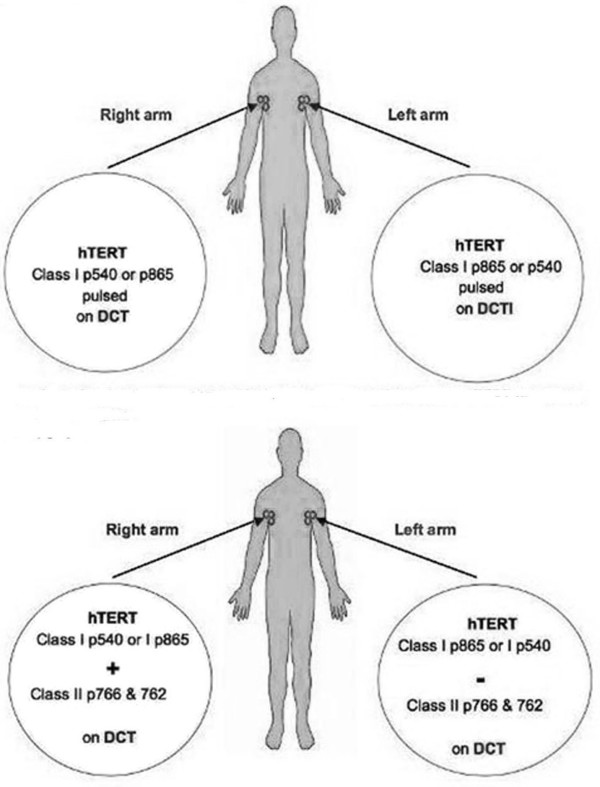
**A. Vaccination design comparing two DC preparations**. DCT and DCTI pulsed with class I epitopes of hTERT; B. Vaccination design comparing two DCT vaccines: DCT pulsed with both class I + II epitopes of hTERT and DCT pulsed with only class I epitopes of hTERT in the same patient.

The trial was adapted from a previously validated protocol by Jonuleit *et al*. for comparing T cell responses to vaccination with mature and immature DCs[43]. It is based on repeatedly inoculating the same lymph node draining region with the same vaccine on each arm of the patient[43]. In our study, each DC preparation (DCT or DCTI) was pulsed with only one type of hTERT peptide (p540 or p865) and both preparations were injected into separate lymph node draining regions every 2–3 weeks. This vaccination design enabled comparison of peptide-specific CD8+T cell responses elicited between DCT and DCTI vaccination protocols (phase I of the study; n = 10; Figure 1A). A similar design was used to compare peptide-specific CD8+T cell responses generated by DCs pulsed with class I hTERT peptide (p540 or p865) alone or with class II cognate help (p766 and p672, phase II of the study; n = 6; Figure 1B). Peptides p766 and p672 are known to be promiscuous[52]. Table 1 shows the HLA class II profiles of the patients inoculated with p766 and p672. This was carried out by the National Blood Service Centre (Sheffield, UK), using the Tepnel Lifecodes Luminex, UK, DNA analysis method.

**Table 1:** HLA class II phenotypes: MHC class II allele phenotyping for patients (L001–L006) who were vaccinated with p766 (DR1, 7, 15) and p672 (DR4, 11, 15) of hTERT.

**Patient**	**HLA class II**
**L001**	DRB1*04, DRB1*15;DQB1*0302/07/08/11, DQB1*06
**L002**	DRB1*04, DRB1*1302/31/34/36/39/41;DRB1*0302/**07**/08/**11**, DQB1*06
**L003**	DRB1*04, DRB1*07;DQB1*0301/09/10/13, DQB1*0303/06/12
**L004**	DRB1*08, DRB1*0301/**15**/16/28/35/40/51/53;DQB1*04, DQB1*06
**L005**	DRB1*03, DRB1*04, DQB1*02, DQB1*0301/09
**L006**	DRB1*15;DRB5*01;DQB1*06

The alleles compatible with these peptides are in bold.

HLA Class II testing was carried out by the National Blood Service Centre, Sheffield, UK. The method for HLA testing was through DNA analysis (Tepnel Lifecodes Luminex, UK).

### DC Preparation

All patients had a temporary apheresis line (Bard, Crawley, UK) inserted under local anaesthesia. Apheresis, using a Kobe apheresis unit, was performed in the Stem Cell Unit, Nottingham City Hospital. The sterile apheresis product was transported to the Rayne Institute, King's College Hospital, London (a registered GMP facility), for vaccine production. The product was washed twice, in MACS Buffer (Miltenyi Biotech). After counting, cells were labelled with anti-CD14+ immunomagnetic beads. CD14+ cells were purified using a paramagnetic filter (Clini Macs-Miltenyi Biotech)(6). The purified CD14+ cells were washed and then incubated in XVIVO-20 (Bio Whittaker, Walkersville, USA) serum-free medium containing gentamycin (100 μg/ml) at a cellular concentration of 3 × 10^5^/ml in 150 ml culture flasks (Nunc, 175 cm^2^, Sigma-Aldrich, UK). Monocytes were cultured in cytokines with purity in excess of 95% (recombinant human IFN-α_A_, carrier free and 97% pure from PBL Biomedical Laboratories, New Jersey, USA; recombinant human IL-4, GM-CSF and TNF-α, carrier free and 95% pure from R&D Systems, Abingdon, UK) with prior approval from the MHRA according to the two protocols. The culture medium was supplemented with IL-4 (500 IU/ml), GM-CSF (500 IU/ml) and TNF-α (110 IU/ml) [DCT] or with (IL-4, GM-CSF, TNF-α and IFN-α (500 IU/ml) [DCTI]. Cytokines and medium were replenished on day 4. On day 7, non-adherent DCs were removed by gentle rinsing, washed and then resuspended in 5 mls of medium. DCs were pulsed with p540 or p865, 40 μg/ml for 4 hours (h). They were then washed once before being cryopreserved in aliquots of 1 ml of XVIVO containing 20% dimethyl-sulphoxide (DMSO, Insource, USA) at a cellular concentration of 1 × 10^6 ^cells/ml.

### Patient Vaccination

Each patient received both types of vaccine at the same time. In every other patient, the DCTI vaccine was pulsed with p540 and the DCT vaccine pulsed with p865. In alternate patients, the DCTI were pulsed with p865 and the DCT pulsed with p540 (Figure 1A). Comparisons were made for vaccinations with or without class II cognate helper epitopes (p766 and p672), by both cognate helper peptides with a different class I peptide in each alternate patient (Figure 1B). DCs were pulsed with class I (40 μg/ml for 4 h) and class II epitopes (40 μg/ml for 4 h) or class I epitopes of hTERT (40 μg/ml for 4 h) only. Vaccines were transported from the Rayne Institute, London to the County Hospital, Lincoln, in dry ice, and thawed immediately prior to administration. Intradermal vaccinations (total 1 ml) were delivered into either the upper or lower limb, or the groin. Each type of vaccine (2 × 10^6 ^DCs/ml) was always administered at the same site. Patients were vaccinated 2 or 3 weekly for 2 to 21 cycles (Mean = 7 cycles), phlebotomy being performed immediately prior to vaccination.

### Delayed Type Hypersensitivity (DTH) Responses

Erythema and/or induration of 10 mm or greater (by callipers) at 48 h following vaccination, at the inoculation site was considered a positive DTH response.

### Tetramer Analysis of Peptide Specific T Cells

Tetramer analysis was performed on patients' peripheral blood mononuclear cells (PBMCs). Tetramers were manufactured by the tetramer facility at the National Institute of Allergy and Immunity, Emery University, USA. Tetramers were conjugated to Phycoerythrin (PE) and shipped at a concentration of 1 mg/ml and the optimum working dilution of the tetramer was determined by serial dilution; 1/125 to 1/150 was found to be optimal. Cells were stained with fluorescein isothiocyanate (FITC)-conjugated anti-CD8 (Sigma Aldrich, UK) and PE-conjugated tetramers for 30 min at 4°C. Cells were washed twice in phosphate buffered saline (PBS) before being fixed in 0.5% paraformaldehyde.

### T2 Cytotoxicity Assays

T2 cells (TAP deficient, HLA-A2.1+) were obtained from the American Type Culture Collection (ATCC) and maintained in Iscove's Modified Dulbecco's Medium supplemented with glutamine, and penicillin and streptomycin (100 IU/ml and 100 μg/ml, respectively, Sigma-Aldrich, UK). Peptide-pulsed T2 cells (10,000), pre-labelled with PKH26 (Sigma-Aldrich, UK), were incubated with mononuclear cells at an effector to target cell ratio of 10:1 for 4 h, in 100 μl of tissue culture medium (TCM). The latter consisted of RPMI 1640 medium (Sigma-Aldrich, UK.), containing penicillin and streptomycin (100 IU/ml and 100 μg/ml, respectively; Sigma-Aldrich, UK) and 10% heat-inactivated (56°C for 1 hr) foetal calf serum (FCS) (Sigma-Aldrich, UK). Following incubation, cells were stained with Annexin-V FITC (BD Pharmingen, UK) and ToPro3 (Molecular Probes, UK) to demonstrate apoptosis and cell necrosis, respectively[53]. Cells were analysed in a flow cytometer. Gating of dot plots on PHK26+ cells allowed separation of target and effector populations. Cytotoxicity assays were done in triplicates, with T2 cells either peptide-pulsed or not.

### SCC-4 Cytotoxicity Assays

Cytotoxicity assays were carried out using a MHC peptide+ (hTERT naturally expressed) cell line SCC-4 (squamous cell carcinoma-4) and incubating with naïve patient PBMCs (n = 7) stimulated with DCTI and DCT in the presence of the 2 peptides p540 and p865, separately, following 3 cycles of in vitro stimulation. The results are included as supplementary data (SCC-4 cytotoxicity assay). Radiated DCTIs and DCTs (10,000 cells) pulsed with p540 or p865, when used to re-stimulate (× 3 times, weekly) naïve patient PBMCs (100,000 cells) were able to generate cells capable of lysing SCC-4 cells. The cytotoxicity was assessed after incubating 10,000 SCC-4 cells (PKH26 prelabelled) with 100,000 PBMCs and incubated for 4 h. Cells were stained with FITC-conjugated Annexin V and ToPro3 (Sigma Aldrich, UK) and cells that were PKH26+, annexin high and ToPro3 high were regarded as dead. The SCC-4 cells were a gift from Prof Theresa L Whiteside, University of Pittsburgh Cancer Centre.

### Immunofluorescent Staining and Flow Cytometry

Expression of mononuclear phenotypic cell surface markers was assessed using FITC-conjugated Lineage cocktail antibodies (CD3, CD14, CD16, CD19, CD20 and CD56; Becton Dickinson Systems, Oxford, UK.), PE-conjugated anti-HLA-DR and CD40, and allo-phyco-cyanin (APC)-conjugated anti-HLA-DR (Pharmingen, UK), Phycoerythrin cyanin-5 (PE-Cy5) conjugated anti-CD83 (Sigma-Aldrich, UK) and PE-Texas red (ECD) conjugated anti-CD86 (Beckman Coulter, UK). The EPICS ALTRA flow cytometer equipped with blue, red, and violet lasers (Beckman Coulter, UK.) was used in the analysis.

### hTERT Peptides

For vaccinations studies, GMP grade hTERT peptides (540ILAKFLHWL548, 865RLVDDFLLV873,766II LTDLQPYMRQFVAHL and 672II RPGLLGASVLGLDDI, Bachem^®^, Germany) were used. Prior to use, peptides were dissolved in DMSO (Insource, USA). DCs were pulsed with peptides for 4 h at a concentration of 40 μg/ml.

### T regulatory Cell (Treg) Analysis

PBMCs at each vaccination time point for N009, N010, L001, L002, L003, L004, L005 and L006 were stained for T reg surface staining with CD4-ECD and CD25-PE (Sigma-Aldrich, UK) was followed by intracellular staining with foxp3-Alexa4 (Pharmingen, UK) by a well established protocol[54]. Lymphocyte region and CD4+ high/side scatter low region were gated onto CD25 and foxp3, double positive quadrant (Figure 10). Total events acquired were 200,000.

### Statistical Methods

Data from groups were analysed using the student t-test for parametric variables. Non-parametric variables were compared using the Mann-Whitney-U test and Wilcoxon sign rank test, and were considered significant if p < 0.05. Statistical tests were performed using SPSS version 16.0 for Mac.

## Results

### Dendritic Cell Phenotype

**Figure 2:**
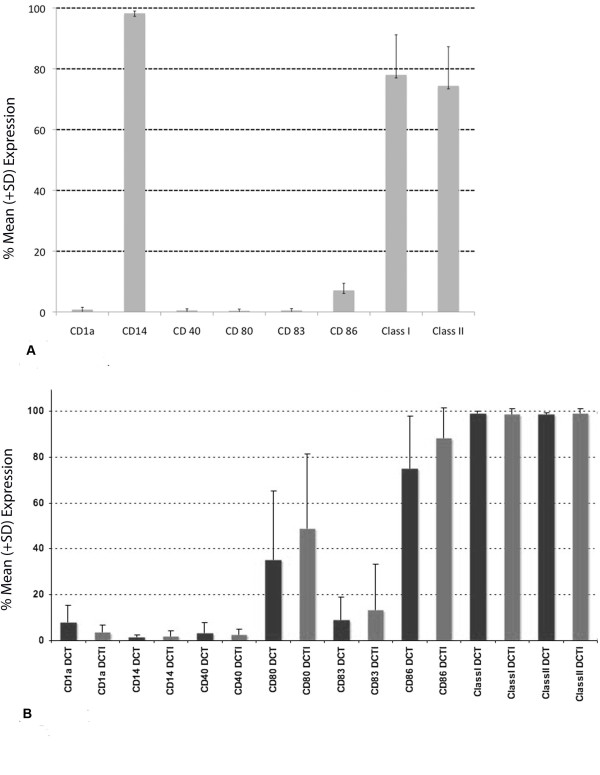
**A. Phenotypic profiles of DC precursor CD14+ monocytes**. Illustrating the absence of DC markers on this monocyte population. B. DC phenotypic profiles: Expression of DC phenotypic surface markers of DCT compared with DCTI preparations (n = 10); see materials and methods for details regarding DC culture conditions. Statistical analysis did not reveal any statistically significant difference between phenotypic markers for DCT and DCTI.

The phenotypic profiles of the precursor monocyte population is illustrated in Figure 2A. Figure 2B summarises the phenotypic profiles of DCs generated *ex vivo *by different processes. DCT and DCTI contained CD14+ cells at substantially lower levels (<5%) than the starting monocyte population. There was upregulation of CD80, CD 86, CD83, CD40, class I and class II with both the DCT and DCTI preparations, when compared with the starting monocyte precursor population. CD40 was not significantly enhanced in either DCT or DCTI with added cytokines, albeit both preparations had substantially increased CD86+ DCs. CD83 expression (a marker of terminally differentiated mature DCs) was less than 10% in the majority (70%) of preparations, even with DCTI. CD80, CD86, CD83, CD40, class I and II did not show statistically significant higher level of expression in DCTI, compared with DCT preparations.

### *In Vivo *Peptide Specific CD8+ T Cell Responses (*Class I Peptides*)

**Figure 3:**
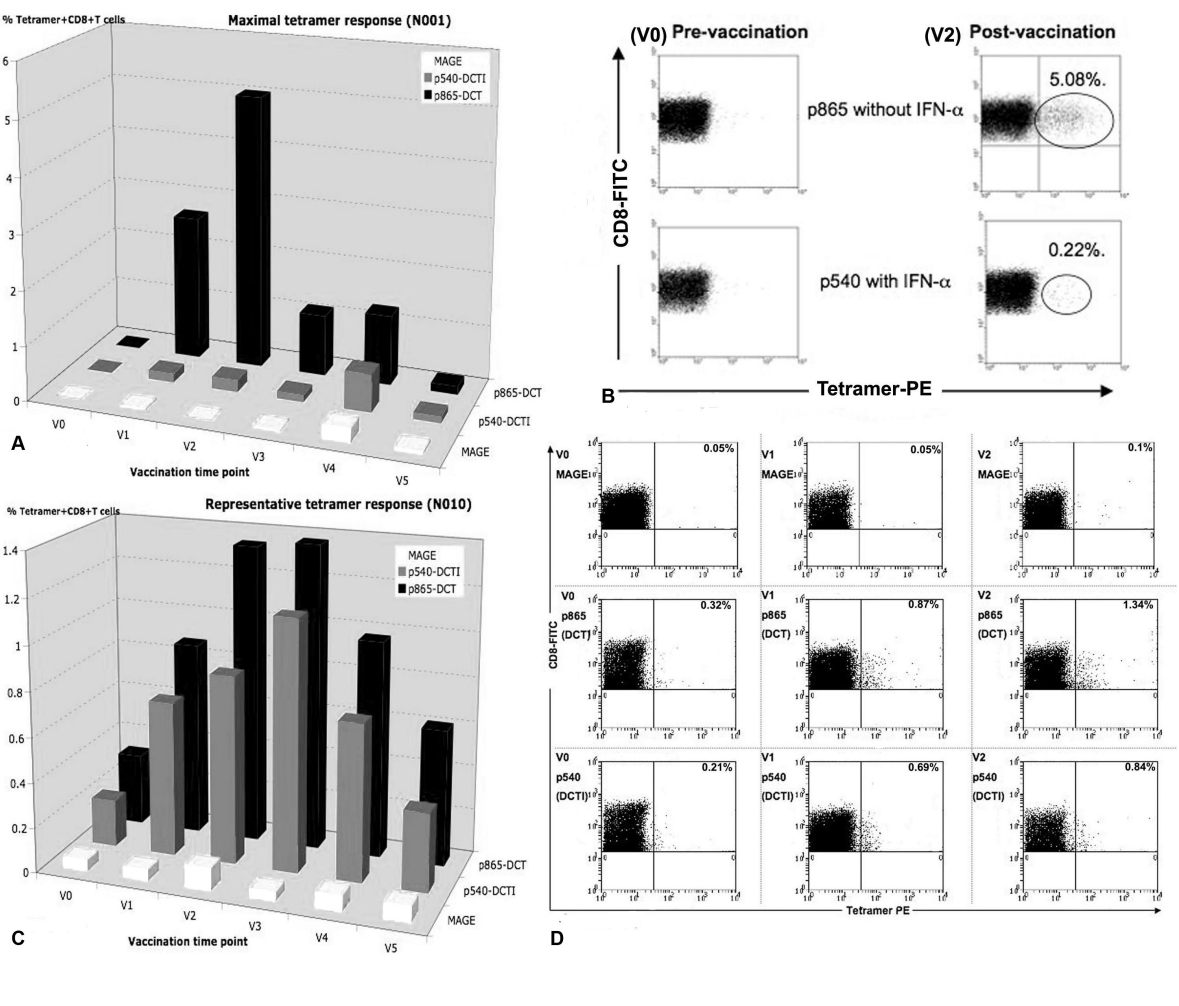
**A. Maximal tetramer response (DCT vs DCTI)**. Time course of tetramer response to vaccination in a patient (N001) who generated the highest level of tetramer+CD8+T cells after 2 courses (V2), compared with baseline (V0). B. Flowcytometry of peak tetramer response: Tetramer flowcytometry plots for N001 at V0 and V2, 150,000 events were acquired and analysed. C. Representative tetramer response (DCT vs DCTI): Time course of tetramer responses to vaccination in a representative patient (N010) who generated equivalent tetramer+CD8+T cell responses to DCT and DCTI. D. Flowcytometry of representative tetramer response: Tetramer flowcytometry plots for N010 at V0, V1 and V2. MAGE-3 was used as the control, non-TAAg;150,000 events were acquired and analysed.

**Figure 4:**
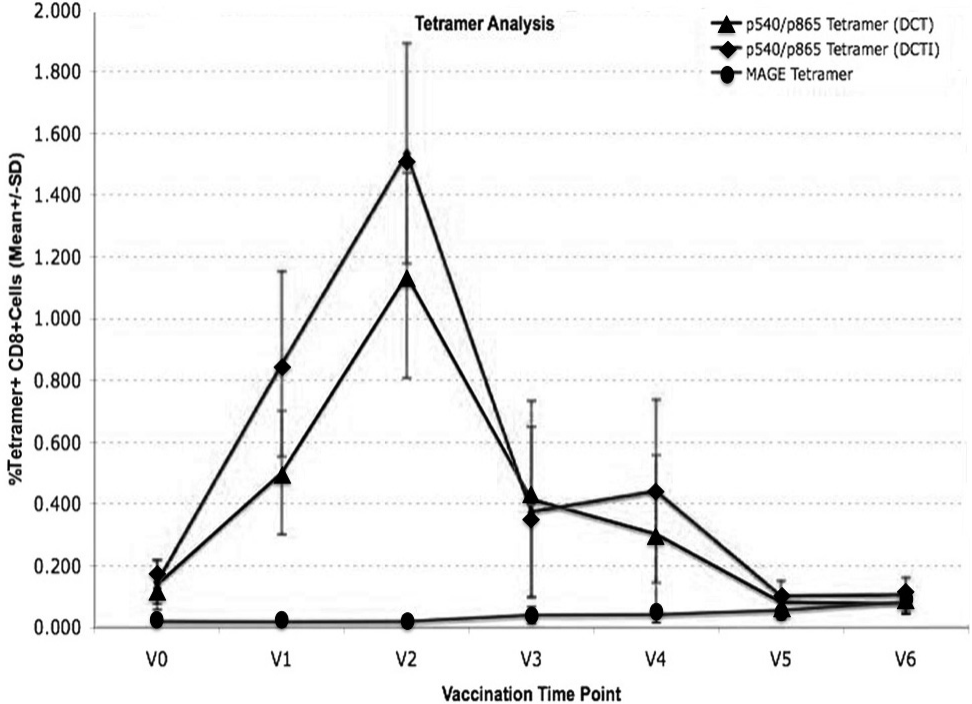
**Tetramer+ CD8+ T cell responses (mean +/- SD) to only class I hTERT pulsed DCs**. Vaccinations with DCT and DCTI in 10 patients. Both vaccines (DCT and DCTI) were equivalent in eliciting CD8+T cell responses and there were no statistically significant differences between DCT and DCTI at any vaccination time point (NS-not significant, p = 0.45, t-test). CD8+T cell tetramer+ response to an irrelevant HLA*A201 MAGE antigen, not used in the vaccination, was measured as a negative control; 150,000 events were acquired and analysed.

**Figure 5:**
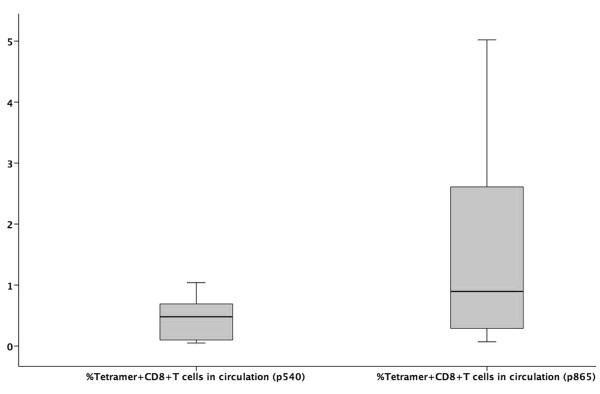
**Box plot comparing tetramer responses to class I hTERT peptide**. Class I peptides of hTERT (p540 and p865) were compared for the efficacy of the tetramer response. hTERT-p865 generated a higher tetramer response compared with hTERT-p540, though this was not statistically significant (p = 0.06. Wilcoxon signed rank test). Values are represented as median(bar), interquartile range (box) and range (whiskers).

Tetramer analysis was performed on PBMCs taken from 10 patients with advanced cancer who had been vaccinated on 2 to 8 occasions (Mean = 4) with DCT and DCTI pulsed with hTERT peptides. Figure 3A and 3B show the time course and flow cytometry plots for a patient (N001) who developed the peak tetramer response to vaccination. Flow cytometry plots are shown for both DC preparations prior to, and after two courses of vaccination. In this particular patient with advanced breast cancer, p865 (DCTI) produced a substantial generation of tetramer+, CD8+, T cells. In fact, the responses generated to the two DC preparations were atypical only in this particular patient. The remaining patients showed the pattern of responses documented in 3C and 3D. Figure 3C and 3D are from a representative patient (N010) and show the comparable magnitude of tetramer responses, elicited in all patients (except N001), to vaccination with DCT and DCTI. The characteristic time course pattern of tetramer+ CD8+ T cell responses was a peak observed after 2–3 courses of vaccination, followed by a gradual tapering of the response to base line levels. Whether this represents failure to mount a continuing optimal response or selective entry of CD8+ T cells into the tumour *milieu *is unclear. There was no tetramer binding to CD8+ T cells using tetramers made with an irrelevant peptide (MAGE-3), which was used as a negative control. Figure 4 shows the mean +/- SD tetramer responses elicited in all of the 10 patients studied. The pattern of response to either class I hTERT peptide was comparable. Both DCT and DCTI vaccines generated equivalent peptide-specific, tetramer+, CD8+ T cell responses (Figure 4). Tetramer+CD8+ responses generated against each class I peptide are shown in Figure 5, though this was not the primary objective of this study. hTERT p865 pulsed-DCs regardless of DC activation protocol (DCT or DCTI) appeared to generate better tetramer responses *in vivo*, compared with p540-pulsed DCs. However, this was only of borderline significance (p = 0.06).

### *In Vivo *Peptide-Specific CD8+ T Cell Responses (*Class I and II Peptides*)

**Figure 6:**
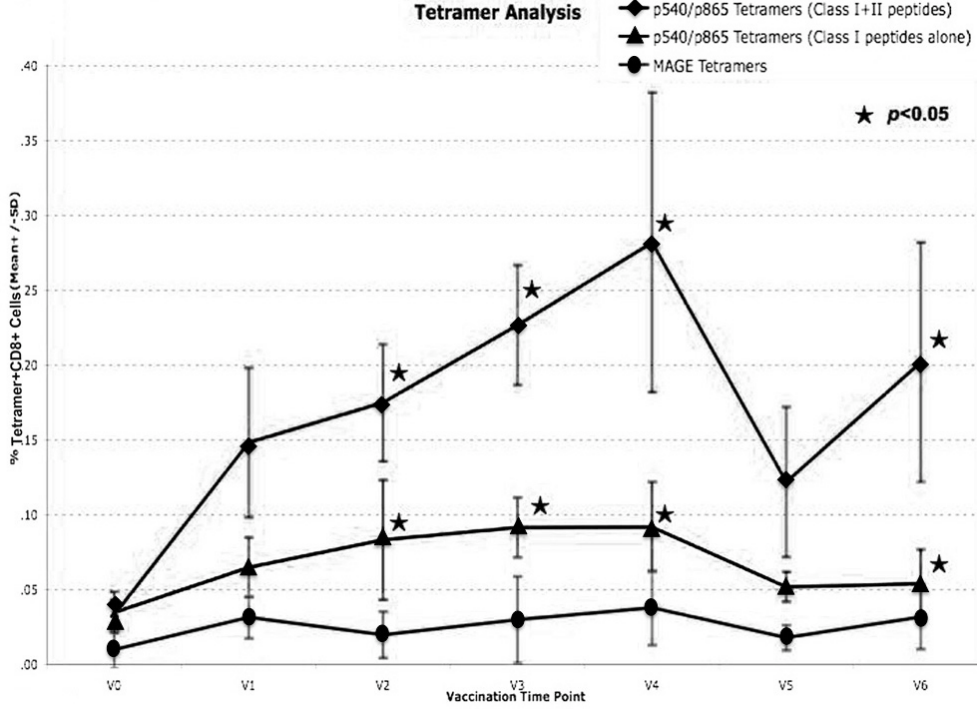
**Tetramer+ CD8+T cell responses (mean +/- SD) to class I ± II hTERT pulsed DCs**. Vaccinations with DCTs pulsed with class I hTERT epitope alone compared with or without class II epitopes in 6 patients. DCTs pulsed with class I+II epitopes showed a strong tendency to enhance tetramer+CD8+T cell responses which were significant (p < 0.05, t-test) at specific vaccination time points V2, V3, V4 and V6 (ie 4 out of the 6 post-vaccination time points). CD8+T cell tetramer+ response to an irrelevant HLA*A201 MAGE antigen, not used in the vaccination, was measured as a negative control; 150,000 events were acquired and analysed.

**Figure 7:**
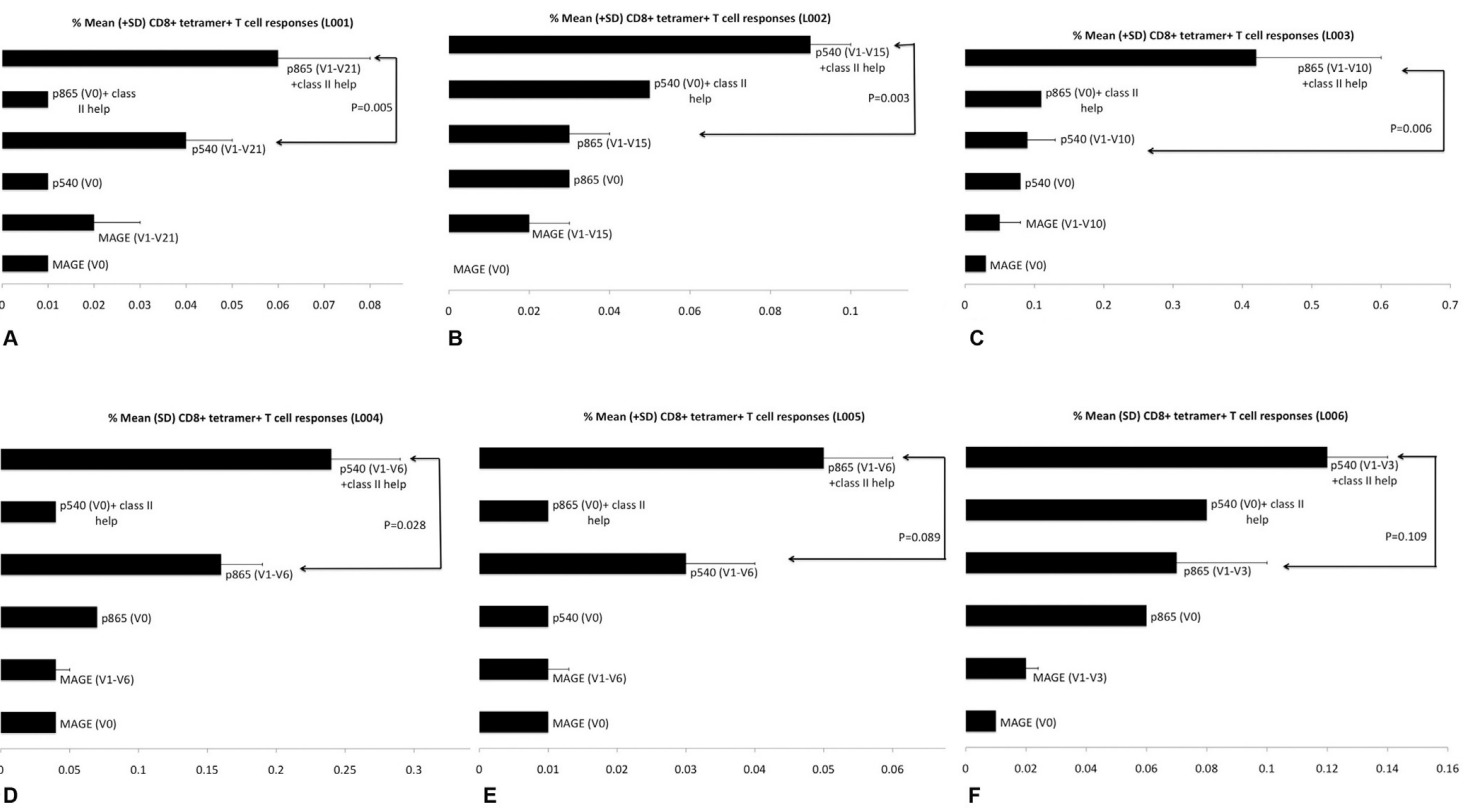
**A, B, C and D.** Post-vaccination tetramer analysis with DCT pulsed with class I ± class II hTERT. tetramer+ CD8+T cell responses (mean +/- SD) to DCTs pulsed with class I hTERT epitope alone compared with or without class II epitopes from patients L001–L004; E and F. Responses were higher and statistically significant (p < 0.05) in patients L001, L002, L003 and L004; Responses were higher with class II epitopes but not significant (NS) statistically in patients L005 and L006 (p = 0.089 and p = 0.109) when analysed using the independent t-test. Each histogram represents either baseline (V0) tetramer response or mean (SD) tetramer responses assessed over multiple time points as indicated in the parethesis (V1-Vx; Vx being the last vaccination time point). CD8+T cell tetramer+ response to an irrelevant HLA*A201 MAGE antigen, not used in the vaccination, was measured as a negative control; 150,000 events were acquired and analysed.

**Figure 8:**
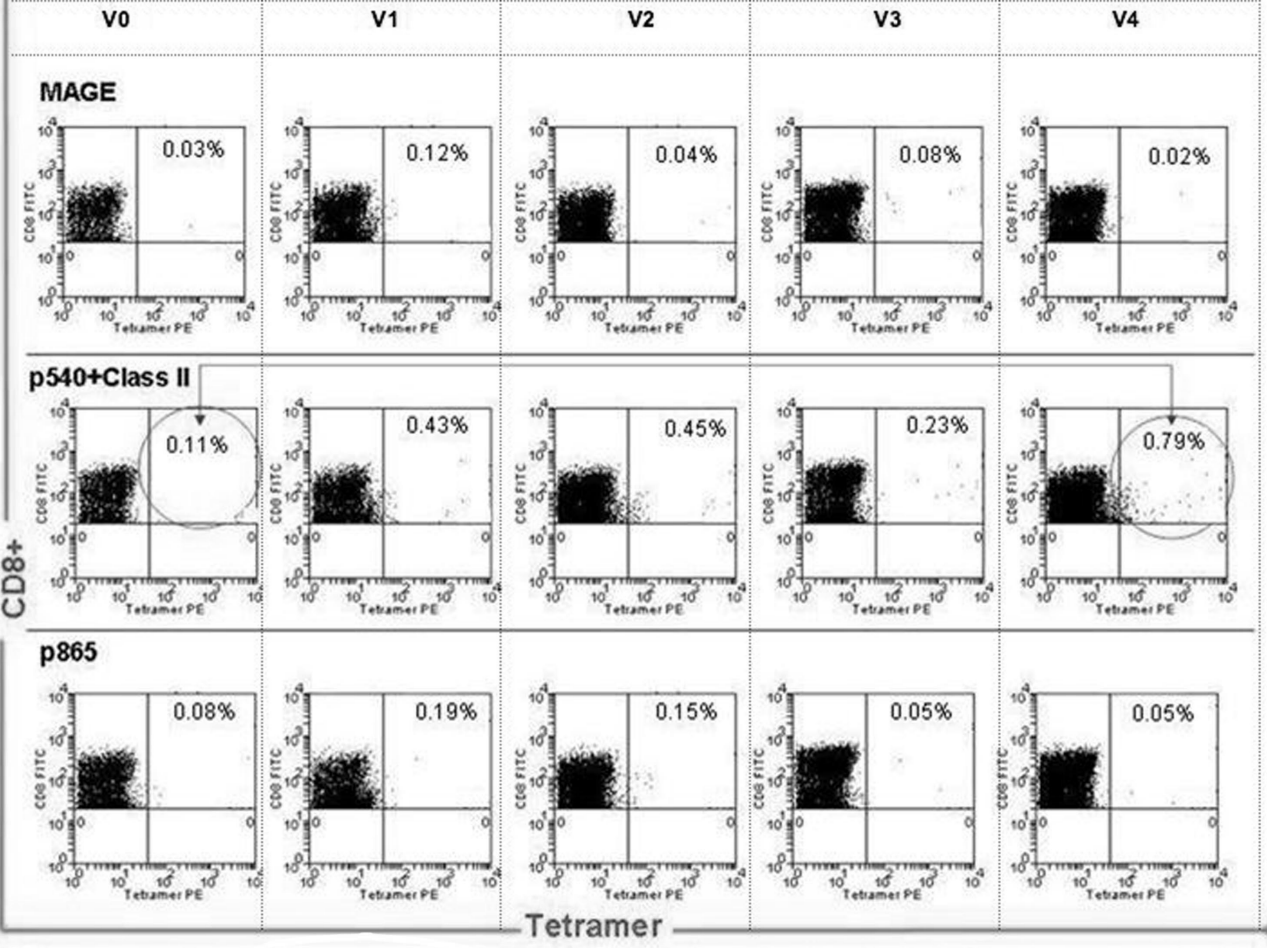
**Representative flowcytometry plots (class I ± II pulsed DCT)**. Tetramer+CD8+T cell responses (in patient L003) elicited from vaccinating with the class I (p865) epitope alone compared with the class I (p540)+ class II epitopes (p766 and p672), through vaccination time points V0–V4 (V0: baseline, V4: following 4^th ^vaccination). The arrow highlights the enhanced response at V4 compared with V0. CD8+T cell tetramer+ response to an irrelevant HLA*A201 MAGE antigen, not used in the vaccination, was measured as a negative control; 150,000 events were acquired and analysed.

Tetramer+ CD8+, T cell responses to hTERT class I epitope peptide, linked with class II cognate help pulsed DCTs, showed a strong tendency for enhancement, with significant differences in 4 out of 6 post-vaccination time points, in comparison with the use of class I peptide-pulsed DCTs alone, as illustrated in Figure 6. Figure 7 demonstrates the enhanced generation of tetramer+ CD8+ hTERT peptide-specific T cell responses with class II cognate helper peptides. This was seen irrespective of the class I peptide (p540 vs. p865) used in the vaccination and in all the 6 individual patients studied. There was a 1.5 to 7 (mean 2.9) fold increase of tetramer+, CD8+ T cells with hTERT class I peptides alone, when compared with CD8+T cell responses to an irrelevant peptide (MAGE-3). This response showed a 4.5 to 11 (mean 7) fold increase with class I and II peptides. Figure 8 shows a representative flow cytometric profile of plots of tetramer+CD8+ T cell responses in patient L003, elicited from vaccinating with a class I epitope alone compared with class I+II epitopes. Table 1 shows that both class II epitopes of hTERT (p766 and p672) used in the study were promiscuous.

### *Ex Vivo *Cytotoxicity of *In Vivo *Generated T Cells

#### T2-cytotoxicity

**Figure 9:**
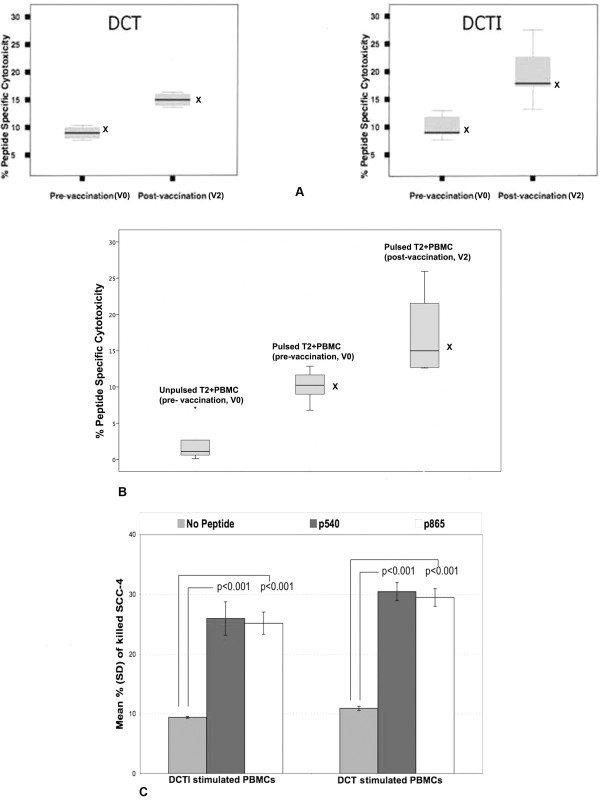
**A. Cytotoxicity against peptide-pulsed T2 cells before and after 2 cycles of vaccination (N001–N010, phase I)**. Enhanced cytotoxicity before and after 2 cycles of vaccination with peptide-labelled T2 cells. Graph on the left shows cytotoxicity of PBMCs generated using DCT vaccine, that on the right shows cytotoxicity of PBMCs generated using DCTI vaccine; 10,000 PKH-labelled T2 events were acquired and analysed. Statistically significant cytotoxicity was observed following 2 vaccinations compared with baseline (X: p = 0.04, Wilcoxon signed rank test). Values are represented as median (bar), interquartile range (box) and range (whiskers). B. Cytotoxicity against peptide-pulsed T2 cells before and after 2 cycles of vaccination (L001–L006, phase II): Cumulative cytotoxicity (mean, SD) before and after 2 cycles of vaccination with peptide labelled T2 cells at an effector to target cell ratio of 10:1. Unlabelled T2 cells were used as a negative control; 10,000 PKH-labelled T2 events were acquired and analysed. Statistically significant cytotoxicity was observed following 2 vaccinations compared with baseline (X: p = 0.028, Wilcoxon signed rank test). Values are represented as median (bar), interquartile range (box) and range (whiskers).C. SCC-4 targeted *ex vivo*cytotoxicity of PBMCs (N001–N007, phase I): Cumulative cytotoxicity of T cells generated by 3 re-stimulations of naïve patient PBMCs *in vitro *by DCT & DCTI with p540, p865 or no peptide; 10,000 SCC-4 cells were incubated with 100,000 PBMCs for this assay.10,000 PKH+SCC-4 events were acquired and analysed. Results are represented as mean (SD). Statistically significant SCC-4 cytotoxicity was observed with DCT and DCTI re-stimulated PBMCs compared with PBMCs restimulated without the class I peptides (X: p < 0.001, t-test), thus showing evidence of cytotoxicity against naturally processed hTERT peptides of SCC-4.

Cumulative cytotoxicity results for all patient samples show that after two cycles of vaccination (the time point associated with the maximal tetramer + CD8+ response, in patients undergoing vaccination with class I peptides only),, *in vitro *cytotoxicity against both peptides was markedly increased, when compared with baseline levels prior to vaccination. Figure 9A shows the cumulative cytotoxicity of PBMCs from patients against the T2 cell line (TAP deficient) before and following 2 cycles of vaccination, comparing DCT and DCTI, for patients (N001–N010). Figure 9C shows the cumulative cytotoxicity of PBMCs from patients against the T2 cell line (TAP deficient) comparing unpulsed, pre-vaccination and following 2 cycles of vaccination (for patients L001–L006). Similarly, significant enhancement of hTERT-specific cytotoxicity was observed following 2 courses of vaccination in L001–L006. Vaccination of all our patients successfully generated not only enhanced tetramer+ CD8+ positive T cells, but also functionally active cytotoxic T cells, capable of destroying targets in a hTERT HLA*A201 class I specific manner.

#### SCC-4 cytotoxicity

SCC-4 cytotoxicity was significantly enhanced (p < 0.001) when peptide loaded DCTs and DCTIs were used to restimulate naïve patient PBMCs (N001–N010) compared with no peptide, as illustrated in Figure 9B. This was a surrogate measure of *in vitro *cytotoxicity against naturally processed peptides of hTERT, as SCC-4 is known to inherently express hTERT peptides on its surface.

### T Regulatory Cell Responses

**Figure 10:**
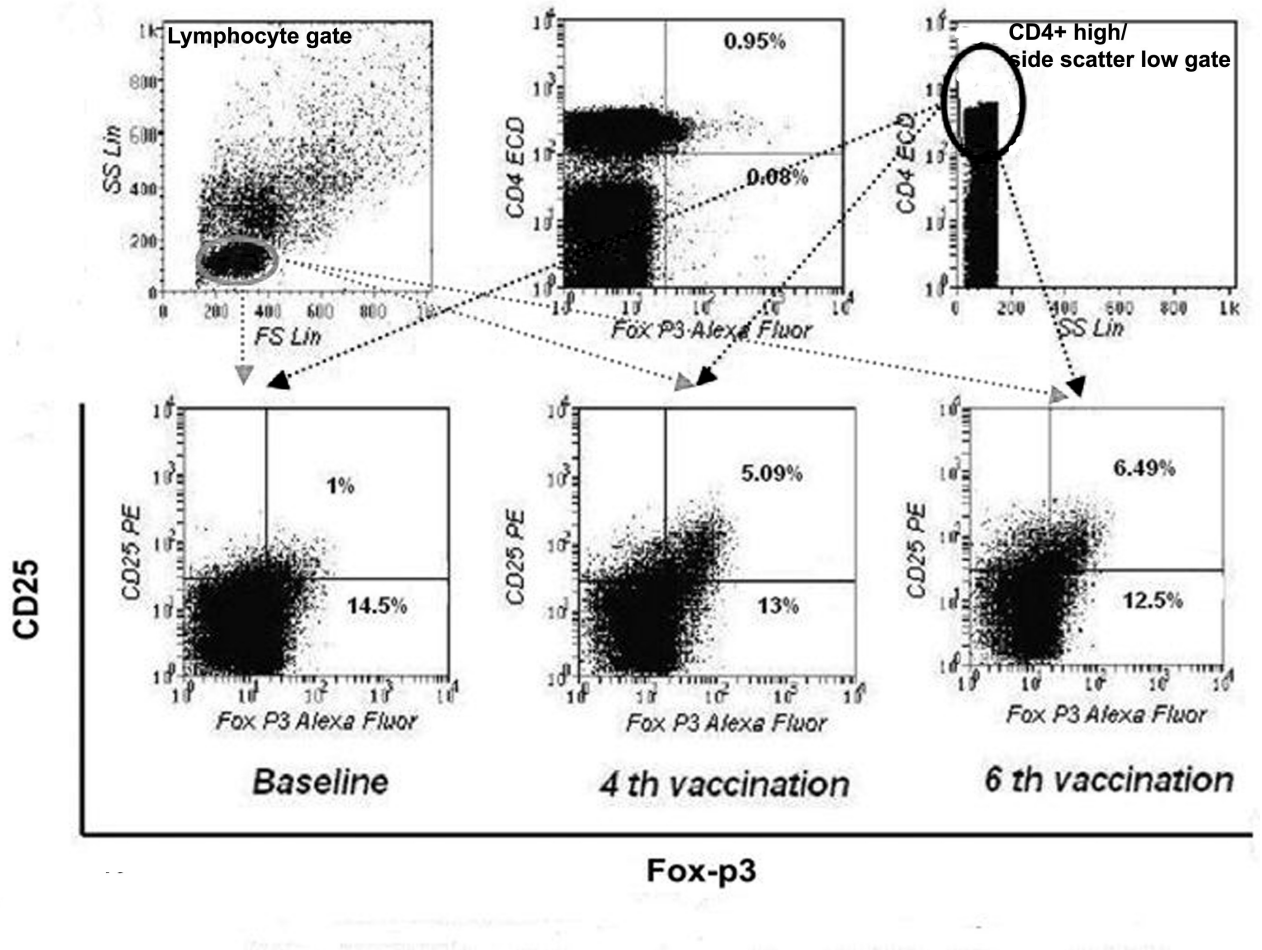
**Representative flowcytometry plots of CD4+CD25+foxp3 high (T regs)**. T regs from patient L001, tracked through vaccination. Data shown are for baseline and just prior to the 4^th ^and 6^th ^vaccinations; 200,000 events were acquired and analysed.

**Figure 11:**
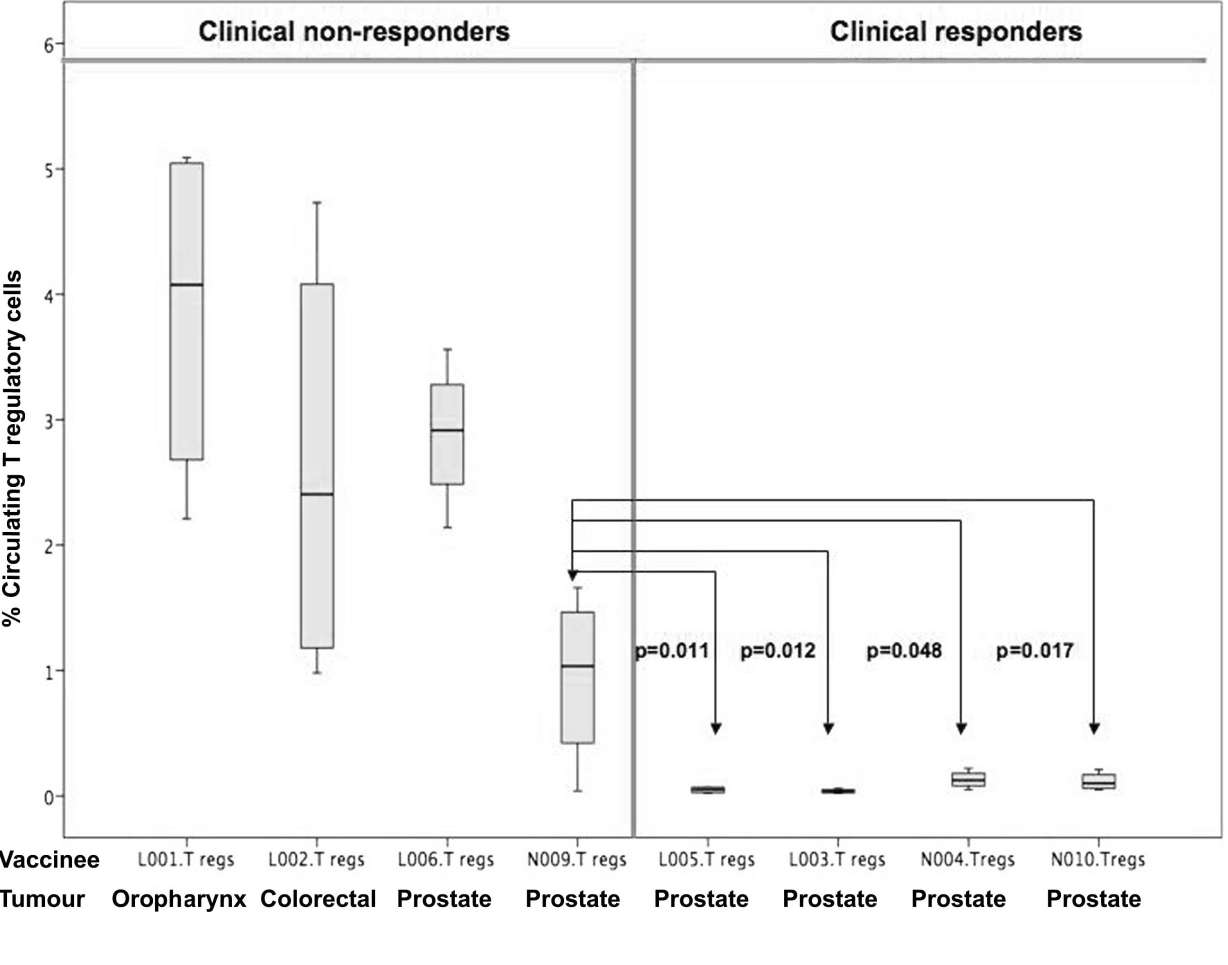
**T regulatory cells from 8 vaccinated patients**. Four patients experienced partial disease resolution (responders); four had stable or progressive disease during vaccination (non-responders). N009 and N010 were vaccinated with only class I peptide pulsed DCs, whilst L001–L006 were vaccinated with both class I and II peptide-pulsed DCs. Lymphocytes were gated on CD4+ high/side scatter low region which in turn was gated onto CD25 and foxp3, double positive quadrant. Total events acquired were 200,000. Values are represented as median (bar), box (interquartile range) and whiskers (range). The patient with the lowest median T reg value (N009, non-responder) was compared with the T reg values of all the responders and was found to be significantly higher (p < 0.05) using the Mann Whitney-U test. Values are represented as median (IQR) for the duration of the course for each patient. The number of vaccinations varied from 4–21. T reg values were measured at each vaccination time point.

T regulatory cell responses were documented and tracked in a total of 8 patients (there being insufficient samples for the remaining patients). A flow cytometry plot of Tregs, representative of that observed in patients experiencing progression of disease, is illustrated in Figure 10. In the 8 patients where Tregs were monitored, it was interesting to note that all patients who experienced disease regression (responders) had a mean circulating T reg level of < 0.5% throughout vaccination (Figure 11), compared with those who had disease progression (non-responders), in which a progressive increase of Tregs was observed during the course of the study.

### Delayed Type Hypersensitivity (DTH) Responses

**Figure 12:**
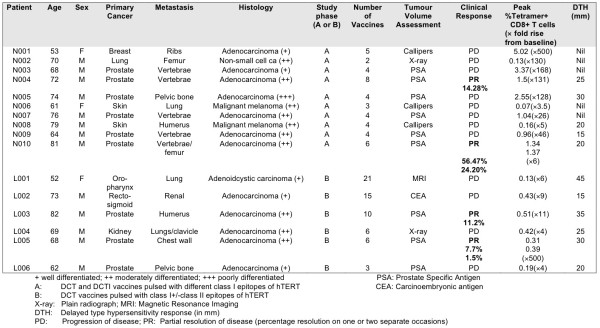
**Clinico-pathological summary**. Relevant clinical and pathological data for all patients who underwent vaccination with hTERT-pulsed DCs.

Five out of 10 patients (50%) in the DCT vs. DCTI group developed DTH responses at the inoculation sites. The average DTH response in this group was 2.2 cm and consisted of erythema or induration whichever was the greatest. All patients (100%) developed DTH responses in the hTERT class I+II vs. class I peptide-pulsed DCs group (Figure 12). The average DTH response was 2.83 cm in this group of patients. There was no obvious correlation between DTH responses elicited and the clinical responses documented. All the 4 patients (prostate cancer) who demonstrated a partial response had a DTH response ≥ 20 mm (Figure 12)

### Clinical Responses

Four out of a total of 16 vaccinated patients experienced favourable clinical responses; 4 prostate cancer patients had partial disease resolution, as assessed by serial monitoring of circulating PSA >10% (Table 2). However, all patients experienced disease progression upon discontinuation of immunotherapy. Circulating prostate specific antigen (PSA) levels were reduced twice in 2 patients and once in the other 2 patients with advanced prostate cancer during vaccination. The fall in PSA was at least 1.5% and upto 56%. The average fall in PSA was 19% (Table 2). Disease stabilization occurred in a patient with colorectal cancer who was inoperable due to loco-regional invasion of the left kidney and adjoining tissues by the tumour. None of the patients received any concurrent therapy during vaccination. All the favourable responders did not have altered renal function or serum albumin levels during the vaccination course, to account for the changes in PSA.

**Table 2:** PSA values: Baseline and reduction of blood PSA levels for patients who showed partial clinical responses following vaccination with the vaccination time point.

**Patient**	**PSA** **baseline** **levels** **(μg/L)**	**Vaccination** **time point (V)**	**PSA** **Post-response** **(μg/L)**	**Vaccination** **time point (V)**	**Percentage reduction**
**N004**	2320	V0	2030	V1	14.28%
**N010**	2798	V1	1218	V2	56.47%
**N010**	3218	V3	2439	V4	24.20%
**L003**	232	V7	206	V8	11.20%
**L005**	465	V2	429	V3	7.7%
**L005**	639	V4	629	V5	1.5%

None of these patients had altered renal function or change in serum albumin during vaccination to account for variations in PSA levels. Normal reference range for PSA is 0–5 μg/L.

### Toxicity

The vaccination was well tolerated by all patients who experienced only grade I toxicity (NCI grade), consisting of flu-like symptoms and fever related to the vaccination itself. However, two patients had jugular vein complications (1 thrombosis, 1 sepsis) related to apheresis line insertion, which required hospitalization. One of these 2 patients died from septic complications, as a result of the of apheresis line insertion.

## Discussion

Vaccination of cancer patients using autologous DCs, pulsed *ex vivo *with peptides/tumour lysates, is a promising strategy, being investigated to treat patients with advanced disease and no further effective therapeutic options available. The best approach has not, as yet, been identified [9]. The current study design was based on a dual vaccination protocol originally used to enable comparisons to be made of the efficacy of activated and immature DCs, using melanoma specific peptide epitopes [43]. The use of hTERT peptides allowed this approach to be used with a wide range of tumour types, as hTERT peptides are expressed on >85% of cancers [55-57].

The optimal stage of DC activation and maturation for generating tumour vaccines is dependent on various components of the vaccination strategy being employed. An effective vaccine requires the capacity to process and present TAAgs, potency in stimulating T cell responses, stability of the phenotype following *in vivo *administration, the ability to migrate to sites of T cell activation and generation of CTLs. Activated and mature DCs results in antigen-specific immunity, while fully immature and inactivated DCs can induce inhibition of the immune response and the generation of tolerance to TAAgs. In contrast, partially mature but activated DCs are optimal for antigen-loading strategies that require internalization and cell processing. CD83 expression, generally, is regarded as a marker of terminally mature DCs. Some studies [58,59] have suggested that antigen loading of relatively immature DCs is superior to antigen loading of terminally mature DCs, as measured by the ability of the DCs to stimulate T cell responses *in vitro*. A recent study, however, has documented contradictory findings[60]. There is, as yet, no general consensus, on this issue and published evidence supports both strategies, using fully or partially mature DCs. We chose to peptide-pulse our DCs on day 7, at the end of the culture period, when the DCs were activated but partially mature. Our assessment of the published evidence that both antigen capture and processing pathways are downregulated in terminally mature DCs and, therefore, peptide pulsing of partially mature DCs was the preferred strategy to use.

Our previous work [36] in breast cancer patients has clearly demonstrated that such DCTs and DCTIs have an optimal antigen uptake capacity and upregulation of CD86, CD40 and class I, but low levels of CD83, as is expected of non-terminally mature DCs. The phenotypic profile of the DCTs and DCTIs generated from monocytes obtained from cancer patients in the present study show similar changes, except for low expression of CD40. As the method of DC generation and phenotypic analysis were similar in these two studies, it is possible that the only differing variables, namely disease profile and tumour load (operable breast cancer in the earlier study [36] and advanced cancers of differing pathological types in the current study) of these two patient groups, was responsible for the different CD40 expressions observed.

The persistence of antigen presentation by the *ex vivo*-loaded DC is a critical parameter determining DC immunogenicity. It takes at least several hours for the injected DCs to reach the lymph nodes and, even then, continued presentation of antigen is necessary for inducing an effective anti-tumour response[61,62]. Since turnover of peptide-MHC complexes is slowed (albeit not abolished upon full DC maturation) especially for peptide-MHC class I complexes, the density of peptide-MHC complexes can be substantially reduced before the *ex vivo *antigen-loaded DCs reach the regional lymph node[63]. Several studies have demonstrated a correlation between antigen persistence in the DC and magnitude of the immune response elicited by vaccination [64-66]. This was another reason to use activated but partially mature DCs in our study.

The first aim of our study, therefore, was to compare (using the dual vaccination protocol) specific cytokine combinations (TNF-α +/- IFN-α) to generate activated and functional DCs from circulating CD14+ monocyte precursors in patients with advanced cancer. The intention was to optimize anti-tumour, peptide-specific CD8+ T cell responses on vaccination *in vivo *and study their effects on CD8+ hTERT-specific T cell responses to class I epitopes (p540 or p865) of hTERT. In the study reported here, autologous DCTs and DCTIs were produced and pulsed with different hTERT class I-restricted peptides, regarded as putative TAAgs. A few studies have concluded that hTERT p540 is not expressed or is cryptic on the surface of tumour cells and that immunization of cancer patients with hTERT p540 leads to the production of T cells that do not recognize tumour cells *in vivo *based on this epitope [67-69].

In contrast to these studies, the ability of our vaccination strategy to generate tetramer+ CD8+T cells specific to p540 of hTERT highlights its possible usefulness as a tumour target. Comparable supporting evidence has been demonstrated by others [70,71]. We observed that the generation of tetramer+ CD8+T cells to hTERT p540 tended to be less efficient compared with hTERT p865. However, in the *in vitro *cytotoxicity assays, both T cells generated by both peptides were equivalent in lysing SCC-4 cells (expressing MHC class I and hTERT).

Tetramer analysis allowed careful documentation and tracking of peptide-specific CD8+ T cells produced as a result of the *in vivo *immunizing activity of each type of DC following vaccination. The clinical and laboratory data presented also shows that both peptides are immunogenic *in vivo *in patients who possess a large tumour load and who probably are immunosuppressed. The tetramer+ CD8+T cells, generated by our vaccination programme, also were functionally effective in killing *in vitro *anti-cancer targets in an hTERT-specific, HLA-A201 restricted manner.

Monocyte-derived DCs, matured with IFN-α and pulsed with viral peptides (HIV, EBV) are found to be very effective in inducing virus-specific T cell responses [29,72]. An *in vitro *study maturing monocyte-derived DCs using IFN-α has demonstrated cross-talk between DCs and NK cells with TNF-α mediating this intercellular communication, thereby, inducing a superior CD8+ T cell response *in vitro *[73]. These results are at variance with earlier studies in which DCs expressed high levels of CD83, when grown in the presence of TNF-α [74]. In all these studies the protocols used to generate IFN-DCs did not utilize IL-4. Generating DCs from CD14+monocytes using GM-CSF, IL-4 and maturing them with TNF-α ± IFN-α (DCT and DCTI) is a novel strategy which was based on our previous work using monocytes from patients with operable breast cancer [36]. DCTIs are superior in phenotype and function compared with DCTs, as shown in a previously published *in vitro *study [36]. In contrast, in our present *in vivo *study, both DCTs and DCTIs were comparable in inducing peptide-specific T cell responses, following vaccination. The cohort of patients in the previous study had early operable breast cancer, whereas the current study included patients with advanced malignancies, with differing pathological types and who had failed to respond to a range of anti-cancer treatments. The differences seen may partly reflect tumour-specific efficacy of the immune response, with DCTIs being superior in early breast cancer patients[36]. Alternately, these differences illustrate the fact that *in vitro *observations pertaining to the efficacy of cancer immunotherapy do not always mirror *in vivo *anti-cancer responses.

We documented low levels of hTERT-specific CD8+T cells (higher than MAGE-specific CD8+T cells) in the circulation prior to vaccination in most patients. This finding is in concordance with that published by Filaci *et al. *(2006), who demonstrated the presence of low numbers of hTERT-specific CD8+T cells in the circulation of cancer patients[75]. However, enhancement of hTERT-specific CD8+ T cells in the circulation, following vaccination in our study were substantially greater than previously documented in the literature, using class I hTERT peptides [42]. Vonderheide *et al *(2004) did not employ a maturation stimulus in the preparation of the autologous DCs used in their studies[42]. Further, *in vitro *stimulation of lymphocytes was required in a related study to achieve the level of tetramer+ T cells observed *ex vivo *in our study [76]. By contrast, we were able to detect them in the circulation of our vaccinated patients without requiring any *ex vivo *culture and expansion of T cell subsets.

Our second aim, was to compare and contrast (using the dual vaccination protocol) the ability of the DC preparation (DCT) pulsed with class I (p540 or p865) and II (p766 and p672) epitopes of hTERT, to generate an enhanced hTERT-specific CD8+T cell response compared with class I epitopes alone on DCs (DCT). The role of CD4+ T cell help in generating and sustaining CD8+T cell responses has long been emphasized and a consensus is emerging that CD4+ T cell help may be particularly important for the proper establishment of CD8+ memory T cells, but may not be essential for generating primary CD8+ CTL responses[77]. Earlier studies suggest that cognate CD4+ T cell help is a prerequisite for optimal activation of CD8+ CTLs for the generation of memory cells [44,45]. In animal models, help was particularly important for the development and function of low avidity CD8+ memory T cells[78,79]. Thus, we hypothesised that concurrent use of CD4+ class II peptides would prolong the T cell response to vaccination, and improve any CTL function generated. Class II peptides from hTERT with promiscuous binding to human HLA-DR have been described. We used a combination of 2 peptides (p672 and p766) which are presented by HLA-DR1, 4, 7, 11 and 15, and are naturally processed from hTERT expressing tumour cells[52]. These haplotypes were present on the peptide-pulsed DCs of our vaccinees in phase II (Table 1). The use of hTERT class II p766 and p672 epitopes, in combination with class I p540 or p865 peptides, to optimize the vaccination protocol has not been previously published. The use of class II peptides derived from the TAAg (rather than exogenous helper antigen such as keyhole limpet haemocyanin) opened the possibility of CD4+ T cell-induced anti-tumour effects, which have been demonstrated in murine model systems with class II negative tumour cells [80,81]. Cognate CD4+ T cell help appeared to significantly augment the CD8+ anti-tumour immune response in all patients vaccinated with class I+II hTERT peptide-pulsed DCs, which may explain the 2/6 (33%) clinical responders (2 transient tumour regressions) in this group, as compared with only 2 clinical responders (20%, both transient tumour regressions) in the 10 patients vaccinated without class II cognate helper epitopes. Tetramer+ CD8+ T cells generated by vaccination with class I peptide-pulsed DCTs and class I+II peptide-pulsed DCTs, were functionally efficient in killing peptide-pulsed T2 targets. To the best of our knowledge, this is the first such finding from vaccination of cancer patients using DCs pulsed with these combinations of class I and II peptides of hTERT.

The study was carefully designed to compare immune responses within individual patients, rather than patient groups. Maintaining the levels of TAAg-specific CD8+ T cells in the circulation is the summation of the capacity to generate them and their possible loss (apoptosis and/or migration into the tumour *milieu*). Enhanced levels of circulating TAAg-specific CTLs does not always correlate with favourable clinical responses in immunotherapy; there appears to be good evidence to the contrary [82]. However, disparity between the numbers of TAAg specific CD8+T cells in metastatic tumours in lymph nodes and in the circulation is well documented [83,84]. The absence of TAAg-specific T cells in the circulation suggests that homing of the tumour-specific T cell populations to tumour sites contributes to the effectiveness of the anti-tumour immunity generated [85]. Unfortunately, due to the limited availability of tumour samples and ethical considerations for invasive biopsies in advanced metastatic disease, we were unable to demonstrate tumour infiltration by hTERT-specific CD8+T cells in regional draining lymph nodes and in metastatic tumour deposits. Nevertheless, the trend observed (low levels of tetramer+ CD8+T cells in the circulation of patients experiencing a partial tumour regression) favours the postulate of tumour infiltration. The possible correlation between low levels of tetramer+CD8+Tcells in the periphery and clinical response as suggested by our observations (Figure 12), requires further investigation.

Moreover, the reductions of PSA levels in the circulation (surrogate marker of anti-cancer responses) observed in 4 of the 16 vaccinated patients with advanced prostatic cancer, who were not receiving any form of curative therapy, is worth noting. Two of our patients with prostate cancer (N010 and L005 in Figure 12) demonstrated PSA reductions on two separate occasions during vaccination. Unlike melanoma, advanced prostate cancers, without any effective anti-cancer therapy, are not known to regress spontaneously. In Uro-oncology, reduction of PSA levels in the circulation are regarded as indicating favourable responses to treatment of metastatic prostate cancer [86]. The PSA level correlates well with advancing clinical stage in untreated patients and is a reliable biological marker of response to treatment [87-90]. Therefore, we consider the reduction of PSA levels in the vaccinated patients as being indicative of a favourable response. However, there are no universally agreed criteria as to the grade of the clinical response based on the fall in PSA.

There were 2 cases of central venous catheter-related complications-N002 (thrombosis) and N011 (sepsis). N001 was treated with anti-coagulants and recovered. However, N011 died due to gram-negative septicemia before vaccination could commence. The frequency of these events are well within the probability of documented occurrence (about 10%)[91,92].

The third aim of our study was to document the presence of T regs and their relationship to clinical responses in vaccinated patients. Low levels (mean < 0.5%) of circulating T regs were found in all clinical responders (prostate cancer patients) who had transient tumour regression. High levels of T regs have been shown to inhibit anti-cancer T cell response in mice [46-48]. To the best of our knowledge, this is the first documentation of a correlation between clinical responses and T regs in cancer patients undergoing hTERT-pulsed DC vaccination. This novel finding is of substantial significance in hTERT-based immunotherapy in particular, and cancer immunotherapy in general.

Studies in man have shown that potent immunosuppressive T regs can be selectively and transiently eliminated and memory T cells increased by pre-treatment with low dose oral cyclophosphamide, using specified therapeutic regimens [93-96]. Such an approach should lead to a sustained and unhindered generation of hTERT specific CTLs by the vaccine, as the proposed dose and frequency of cyclophosphamide to be used has no detrimental effects on the remaining T cell subsets of lymphocytes [93-96]. Denileukin diftitox (Onzar) is a recombinant protein comprising IL-2 fused with the alpha chain of diphtheria toxin, (DAB389+IL-2), capable of transiently eliminating T regs. Phase II/III clinical trials, involving stage IV cutaneous T cell lymphoma and Non-Hodgkin's lymphoma patients, has demonstrated intravenous denileukin diftitox inducing disease regression (partial and complete in 20–30% of patients). This is due to inhibition of protein synthesis in cells expressing high and intermediate affinity IL-2 receptors, thereby, reducing the T reg (CD4+ CD25+foxp3+) population [97-101]. A tumour vaccination trial, using MAGE and MART peptides to directly vaccinate stage IV melanoma patients who were pre-treated with denileulin diftitox, exhibited prominent peptide-specific CTL responses with concurrent reduction of T regs [50]. In light of our T reg findings and the above studies, we suggest that T reg monitoring and abrogation are potentially useful strategies to both predict and induce beneficial clinical responses in hTERT-based anti-cancer immunotherapy.

In summary, the data presented demonstrates for the first time that the *ex vivo *generation of optimally-activated DCs (DCT), preferentially pulsed with class I+II peptides, were able to induce hTERT-specific CD8+CTL generation *in vivo*. IFN-alpha, when added simultaneously with GM-CSF, IL-4 and TNF-alpha, did not appear to induce a superior CD8+ T cell response, whereas cognate help for vaccinating DCs appeared to augment CD8+ T cell responses. Clinical responses do not always correlate with the levels of tetramer+CD8+T cells in the circulation. However, circulating levels of T regs may predict those likely to have a beneficial clinical response. Our findings will help to establish novel strategies designed to produce clinically effective and immunologically relevant vaccination protocols.

## Competing interests

The authors declare that they have no competing interests.

## Authors' contributions

OE, FF, NH, MMA, RAR, AJM, SS, JME, ME: conception and logistics of the study. MMA, CV, SS, RAR, SC AJM: vaccination of patients, acquisition of samples and generation of data. MMA, AJM, NRH, JB, FF: preparation of the vaccine. MMA, AJM, RAR, OE: critically drafting and reviewing the manuscript, including statistical analysis. MMA, AJM, JME, ST: recruitment of patients into the study and reviewing the manuscript. All authors read and approved the final manuscript.
